# Crystal structure of the WD40 domain dimer of LRRK2

**DOI:** 10.1073/pnas.1817889116

**Published:** 2019-01-11

**Authors:** Pengfei Zhang, Ying Fan, Heng Ru, Li Wang, Venkat Giri Magupalli, Susan S. Taylor, Dario R. Alessi, Hao Wu

**Affiliations:** ^a^Department of Biological Chemistry and Molecular Pharmacology, Harvard Medical School, Boston, MA 02115;; ^b^Program in Cellular and Molecular Medicine, Boston Children’s Hospital, Boston, MA 02115;; ^c^Medical Research Council Protein Phosphorylation and Ubiquitylation Unit, College of Life Sciences, University of Dundee, DD1 5EH Dundee, Scotland, United Kingdom;; ^d^Department of Chemistry and Biochemistry, University of California, San Diego, La Jolla, CA 92093;; ^e^Department of Pharmacology, University of California, San Diego, La Jolla, CA 92093

**Keywords:** LRRK2, WD40, crystal structure, Parkinson’s disease

## Abstract

Leucine-rich repeat kinase 2 (LRRK2) is a large multidomain protein with both a Ras of complex (ROC) domain and a kinase domain (KD) and, therefore, exhibits both GTPase and kinase activities. Human genetics studies have linked LRRK2 as a major genetic contributor to familial and sporadic Parkinson’s disease (PD), a neurodegenerative movement disorder that inflicts millions worldwide. The C-terminal region of LRRK2 is a Trp-Asp-40 (WD40) domain with poorly defined biological functions but has been implicated in microtubule interaction. Here, we present the crystal structure of the WD40 domain of human LRRK2 at 2.6-Å resolution, which reveals a seven-bladed WD40 fold. The structure displays a dimeric assembly in the crystal, which we further confirm by measurements in solution. We find that structure-based and PD-associated disease mutations in the WD40 domain including the common G2385R polymorphism mainly compromise dimer formation. Assessment of full-length LRRK2 kinase activity by measuring phosphorylation of Rab10, a member of the family of Rab GTPases known to be important kinase substrates of LRRK2, shows enhancement of kinase activity by several dimerization-defective mutants including G2385R, although dimerization impairment does not always result in kinase activation. Furthermore, mapping of phylogenetically conserved residues onto the WD40 domain structure reveals surface patches that may be important for additional functions of LRRK2. Collectively, our analyses provide insights for understanding the structures and functions of LRRK2 and suggest the potential utility of LRRK2 kinase inhibitors in treating PD patients with WD40 domain mutations.

## Significance

Parkinson’s disease (PD) is a chronic, progressive movement disorder that affects nearly 7 million people globally and 1 million people in the United States. Although there are pharmacological and surgical interventions to treat the symptoms of PD, no targeted therapeutics that reverse the effects of the disease are currently available. Since identification of the first LRRK2 mutations in PD patients in 2004, more than 40 mutations have been found in both familial and sporadic forms of PD. Because of the association of LRRK2 to PD, the structure of the WD40 domain dimer of human LRRK2 presented here will help elucidate the pathogenesis of certain WD40 mutations and provide structure-based template for potential therapeutic interventions.

Parkinson’s disease (PD) is a severe neurodegenerative disorder that often inflicts older people, for which there is neither an objective test for diagnosis nor an effective cure. About 1–2% of the population above age 65 in the world live with PD (1). The cause of PD is unknown, but current research links it to age and certain genetic mutations. To date, mutations in leucine-rich repeat kinase 2 (LRRK2) represent a major genetic contributor to familial and sporadic PD (2–4). The exact physiological function of LRRK2 is not clear although it has been shown to interact with many partner proteins such as microtubules and apoptotic pathway players and contribute to many cellular processes (5, 6). For examples, LRRK2 regulates neurite growth and neurons that express PD-associated LRRK2 mutations show a progressive reduction in neurite length and branching (5, 7). These neurons additionally display phospho-tau–positive inclusions and ultimately undergo apoptosis (5, 7).

**Fig. 1.**
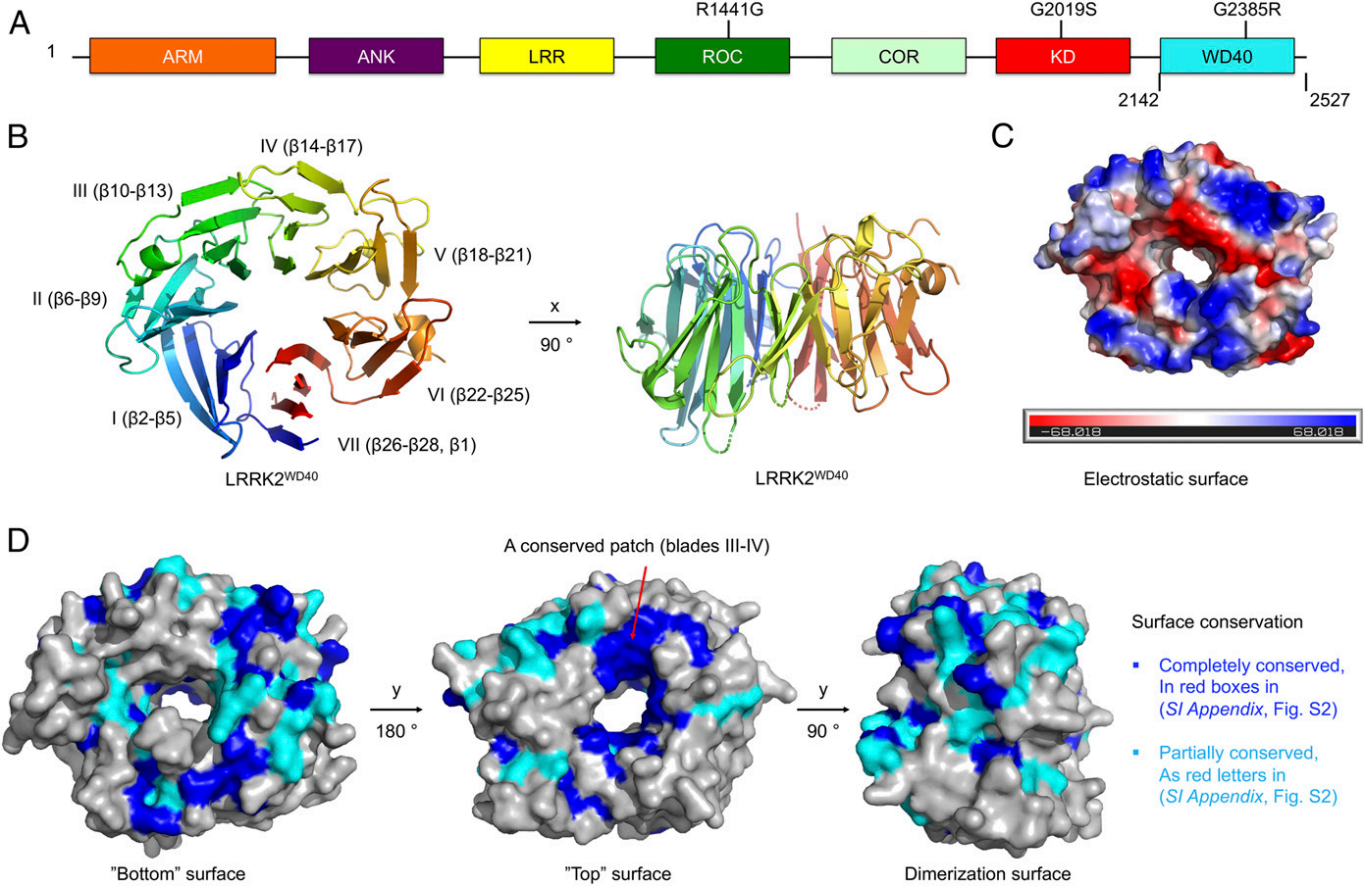
Structural overview. (*A*) Domain organization of LRRK2. The residue boundary of the WD40 domain and locations of three recurrent disease mutations are marked. (*B*) Ribbon diagram using rainbow gradient color from blue at the N terminus to red at the C terminus. The locations of the seven blades are marked. Two views are shown. (*C*) Electrostatic surface diagram showing a prominent hole in the middle of the structure. (*D*) Mapping of sequence conservation onto the WD40 domain structure. The view of the bottom surface is at the same orientation as *B, Left*.

LRRK2 is a large multidomain protein with 2,527 amino acids (286 kDa) and consists of armadillo repeats (ARM), ankyrin repeats (ANK), leucine-rich repeats (LRR), Ras of complex (ROC), C-terminal of ROC (COR), kinase domain (KD), and the Trp-Asp-40 (WD40) domain (5) (Fig. 1*A*). LRRK2 has bienzymatic activities of GTPase and kinase. The kinase activity is generally believed to be important in the pathogenesis of PD (7). The most frequent mutation, G2019S in the kinase domain, increases the kinase activity by about twofold, and GTP binding to the ROC domain may regulate kinase activity (5, 8–10). The crystal structure of ROC bound with GDP and Mg^2+^ showed a domain-swapped dimeric architecture (9). However, subsequent biochemical studies suggest that the ROC domain is a stable and catalytically active monomer in solution, suggesting that the domain swapping may be an artifact of construct or crystallization (11). Consistently, cellular imaging showed that LRRK2 is predominantly monomeric throughout the cytosol but can also form oligomers (12). A recent low-resolution structural model of full-length LRRK2 dimer was reported by negative-staining EM and other methods, in which the ROC and COR domains were interpreted as mediating the dimerization (13, 14). In addition, crystal structure of a ROC-COR construct of Roco, a prokaryotic homolog of LRRK2, revealed a COR domain mediated dimer (15), and full-length Roco undergoes dynamic monomer-dimer transition during GTP turnover (16). Therefore, dimerization is a central question in LRRK2 structure and function.

The C-terminal WD40 domain of LRRK2 is required for LRRK2-induced neurotoxicity (17, 18) and has been shown to mediate the interaction of LRRK2 with microtubules and synaptic vesicles (19, 20). Many PD mutations enhance the microtubule association of LRRK2, forming filamentous aggregates in cells (19). A WD40 domain variant, G2385R, which is a risk factor for PD in ethnic Chinese and/or Asian patients, with an unknown mechanism of action (21, 22), correlates with an altered strength and quality of interactions to synaptic vesicles, resulting in perturbed vesicular trafficking (20, 23). Unlike G2019S with enhanced kinase activity, one study has reported that the G2385R mutation may result in partial loss of kinase function (24), suggesting a pathogenic mechanism different from kinase-activating LRRK2 mutations (25). Additional WD40 domain variants have also been reported as associated with PD risk (26–31).

Here, we report the crystal structure of human LRRK2 WD40 domain determined at 2.6-Å resolution. The structure reveals a canonical seven-bladed WD40 fold and a surprising dimeric state in the crystal. We confirmed that isolated WD40 domain forms concentration-dependent dimers in solution and that structure-based interface mutations mainly disrupt the dimerization. Notably, the WD40 domain mutation G2385R is located at the dimerization interface and compromised dimerization. In contrast to previously identified reduction in kinase activity, our data on Rab10 phosphorylation indicated an enhanced kinase activity, supporting the overarching hypothesis that pathogenesis of PD is mediated through hyperactivation of LRRK2. Similar to G2385R, two structure-based mutants H2391D and R2394E at the WD40 dimerization interface also increased LRRK2 kinase activity. We further mapped sequence conservation onto the WD40 domain structure and found surface patches that may be important for additional functions of LRRK2.

## Results

### Overall Structure of the Human LRRK2 WD40 Domain.

We expressed the WD40 domain of human LRRK2 (residue 2142–2527) in insect Sf9 cells. The protein was purified, and diffracting crystals were obtained. We initially attempted molecular replacement structure determination of the LRRK2 WD40 domain using multiple known WD40 domain structures in the Protein Data Bank (PDB). No viable solution was obtained, likely because of the weak homology between the LRRK2 WD40 domain and WD40 domain structures in the PDB. We therefore searched for heavy atom derivatives using soaking of preformed native crystals. After screening 13 heavy atom compounds (*SI Appendix*, Table S1), diffraction data collected on a crystal soaked with transplatinum (II) diammine dichloride gave sufficient anomalous signals at the wavelength of 0.98 Å, which is a high energy remote wavelength of the platinum L-III edge. A total of three platinum sites were identified (*SI Appendix*, Fig. S1*A*). We determined the WD40 domain crystal structure using the single wavelength anomalous diffraction (SAD) method and refined it to 2.6-Å resolution (*SI Appendix*, Table S2). The ordered part of the structure extends from residue 2,142 to residue 2,498 and has a few gaps, and the last 30 residues in the construct are disordered.

The structure shows a canonical seven-bladed WD40 domain architecture (Fig. 1*B*), with a prominent hole in the middle when viewed from the top (Fig. 1*C*), within which the platinum compound molecules are bound (*SI Appendix*, Fig. S1*A*). The electrostatic potential plot reveals a highly charged surface with positively and negatively charged patches (Fig. 1*C*). A structural homology search in DALI (32) revealed that it is most similar to the WD40 domains in the mRNA export factor Rae1 (PDB ID code 4OWR) (33), the apoptotic protein Apaf-1 (PDB ID code 3SHF) (34), the anaphase promoting complex (APC) protein CDH1 (PDB ID code 5A31) (35), and the coatomer subunit β′-COP (PDB ID code 4J84) (36) (*SI Appendix*, Fig. S1 *B*–*E*). All blades comprise four anti-parallel β-strands. The first six blades run from β2–β5, β6–β9, β10–β13, β14–β17, β18–β21, and β22–β25 continuously, and the last and seventh blade contains β26–β28 and β1 from the N-terminal end to complete the fold, forming a stabilized ring-like structure (Fig. 1*B* and *SI Appendix*, Fig. S2).

LRRK2 WD40 domain sequences from different species are aligned and compared (*SI Appendix*, Fig. S2). Previous structural and biochemical studies have indicated that one surface (top) of the WD40 domain is more frequently used for protein–protein interactions than the opposing surface (37), and a highly conserved surface on a β-propeller is almost always involved in protein–protein interactions (38). We therefore mapped sequence conservation onto the WD40 domain structure of LRRK2 (Fig. 1*D*). This exercise revealed surface patches that are completely or partially conserved across the aligned species. In particular, there is a relatively large conserved patch on the top surface of the LRRK2 WD40 domain formed by residues in blades III and IV (Fig. 1*D*), suggesting additional functions of the domain in LRRK2 biology. In comparison, the dimerization surface is not as conserved (Fig. 1*D* and below).

### Dimerization of the WD40 Domain.

**Fig. 2.**
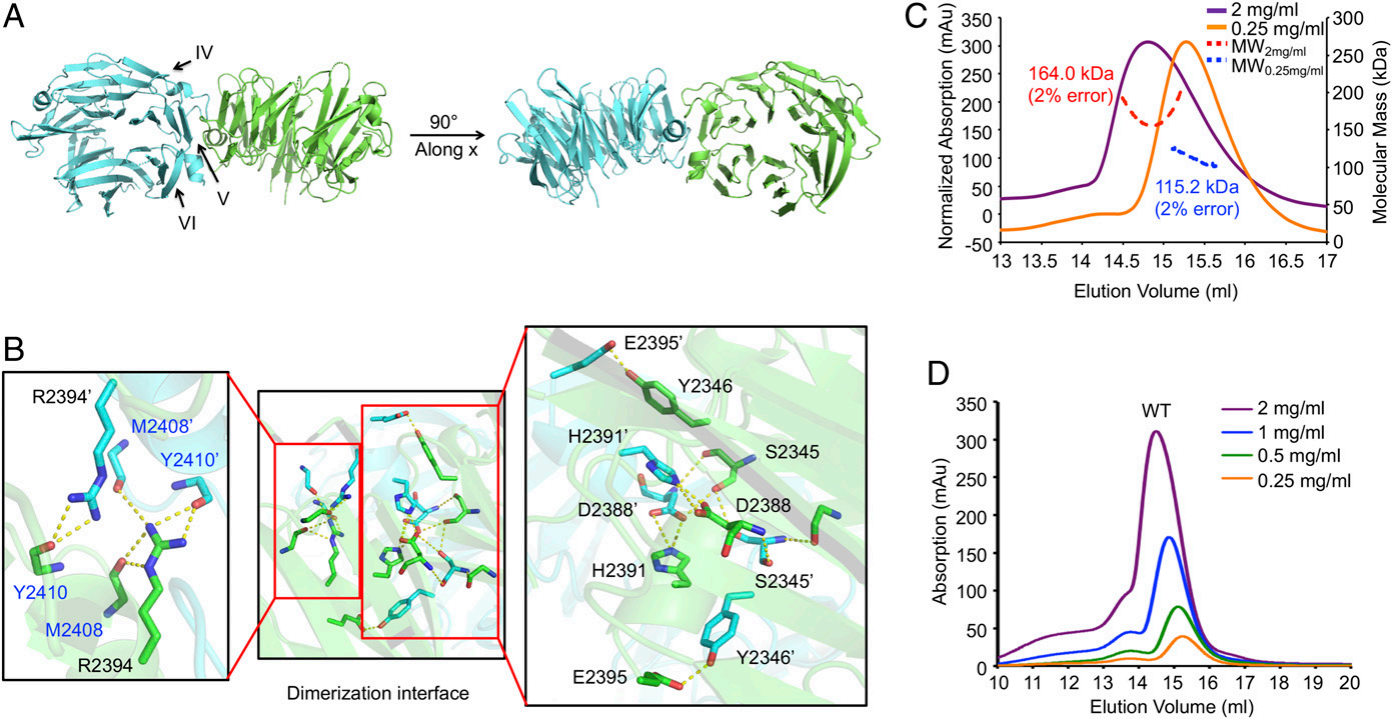
Dimerization of the WD40 domain of LRRK2. (*A*) Ribbon diagram of the dimer in two orientations with the subunits in green and cyan, respectively. (*B*) Detailed interactions at the dimer interface (*Middle*) with enlarged views at *Left* and *Right*. The residues in the symmetric subunit are marked with apostrophe symbol. The residues with main chain atom interactions are shown in blue. (*C*) MALS measurements of WD40 domain of LRRK2 at 2 mg/mL (red) and 0.25 mg/mL (blue), respectively. The absorption values at the left for the 0.25 mg/mL sample were multiplied 8× to scale with the absorption for the 2 mg/mL sample. (*D*) Concentration-dependent dimerization of the WD40 domain. The concentrations of the injected samples are shown, showing the progressive delay in elution at lower concentrations.

There are two WD40 domain monomers in the crystallographic asymmetric unit, which are related by an almost perfect twofold axis (176.3°) (Fig. 2*A*). The interactions are centered at blade V but also involve the surrounding structures as well as blade IV and VI (Fig. 2*A* and *SI Appendix*, Fig. S2). The dimerization interface is large and buries approximately ∼1,200 Å^2^ surface area per monomer. Residues that bury more than 40 Å surface areas are marked (*SI Appendix*, Fig. S2). In addition to surface area burial, a number of potential hydrogen bonding and salt bridge interactions exist, among D2388 of one subunit and H2391 and S2345 of the symmetric subunit, among R2394 of one subunit and main chain atoms of M2408 and Y2410 of the symmetric subunit, and between E2395 of one subunit and Y2346 of the symmetric subunit (Fig. 2*B*).

Given the dimeric association of the WD40 domain in the crystal, we determined whether it also forms dimers in solution. We used multiangle light scattering (MALS) to measure the molecular mass of the MBP-tagged LRRK2 WD40 domain as it eluted from an in-line size exclusion column. The calculated molecular mass of an MBP-tagged LRRK2 WD40 domain monomer is roughly ∼83 kDa. The MALS measurement showed that the LRRK2 WD40 domain peak when injected at 2 mg/mL corresponded to 164.0 kDa (2% error) in molecular mass, corresponding to a dimer (Fig. 2*C*). Therefore, the WD40 domain dimer observed in the crystal is also a bona fide dimer in solution, instead of a crystallization artifact. Further gel filtration chromatography analysis at different concentrations of the LRRK2 WD40 domain showed that it is a dynamic, concentration-dependent dimer, with the peak shifting to later elution positions when serially diluted from 2 mg/mL to 0.25 mg/mL (Fig. 2*D*). In particular, the measured molecular mass of the LRRK2 WD40 domain peak when injected at 0.25 mg/mL was 115.2 kDa (2% error), smaller than a dimer and closer to a monomer of MBP-tagged LRRK2 WD40 (Fig. 2*C*).

### Structure-Based Mutations and PD-Associated Variants in the WD40 Domain.

**Fig. 3.**
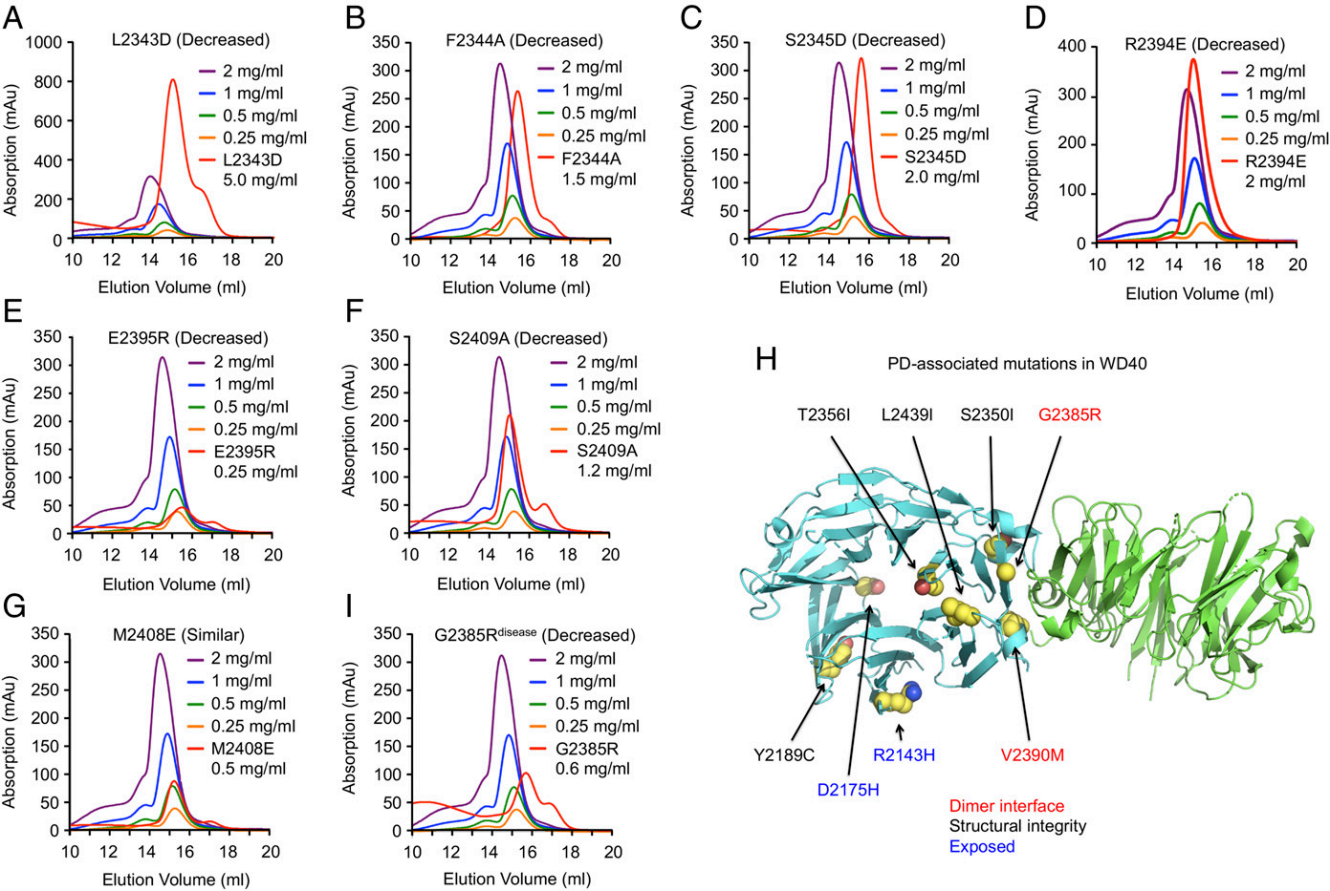
Effects of structure-based and PD-associated mutations in WD40 domain dimerization. (*A*–*G*) Elution positions of structure-based mutants in comparison with the WT WD40 domain. Decreased: decreased dimerization; Increased: increased dimerization; Similar: no change in dimerization. (*H*) Ribbon diagram of WD40 domain dimer with PD-associated mutations highlighted as balls with carbon atoms in yellow, nitrogen atoms in blue, and oxygen atoms in red. These mutations are labeled in red if the mutations occur at the dimerization interface, black if the mutations are on buried residues, and blue if the mutations are on exposed residues away from the dimerization interface. (*I*) Elution position of the G2385R disease mutant in comparison with the WT WD40 domain.

We next identified residues that may be important for dimerization based on the structure (*SI Appendix*, Fig. S2) and investigated whether mutations on these residues affect the dimerization of the LRRK2 WD40 domain in solution (Fig. 3 *A*–*G* and *SI Appendix*, Table S3). Using gel filtration chromatography and matched with concentrations, WD40 domain mutants L2343D, F2344A, S2345D, R2394E, E2395R, and S2409A were shown to interfere with dimerization, while M2408E had little influence on dimerization. The D2388K and H2391D mutants did not express well. Consistent with the above analysis, the gel filtration elution positions of the R2394E mutant showed delays relative to the wild-type (WT) at all four tested concentrations (*SI Appendix*, Fig. S3*A*).

A number of variations in the WD40 domain have been reported in PD (5, 6) (www.molgen.ua.ac.be/PDmutDB/) (Fig. 3*H*). Y2189C (26), T2356I (27), and L2439I (28) are buried and may disrupt the structural integrity. D2175H (28) and R2143H (29) are mutations on exposed residues and may influence potential interactions intramolecularly and with neighboring LRRK2 molecules or other proteins. G2385R (21, 22) and V2390M (30) mutants are on residues at the dimer interface. When assayed for dimerization using purified proteins, both G2385R and V2390M disrupted dimerization of the LRRK2 WD40 domain (Fig. 3*I* and *SI Appendix*, Fig. S3*B* and Table S3). Similarly, D2175H, T2356I, and L2439I also compromised dimerization likely because they altered the protein structure, while Y2189C did not affect the dimer interaction (*SI Appendix*, Fig. S3 *C*–*F* and Table S3). The R2143H variant did not express well. A recent study reported three additional WD40 variants N2308D, N2313S, and S2350I in the mainland Chinese population (31). While residues N2308 and N2313 are both disordered in the structure, S2350 is close to the dimerization interface and may also affect WD40 domain dimerization (Fig. 3*H*).

### Effects of LRRK2 WD40 Dimerization Mutations on Rab10 Phosphorylation.

**Fig. 4.**
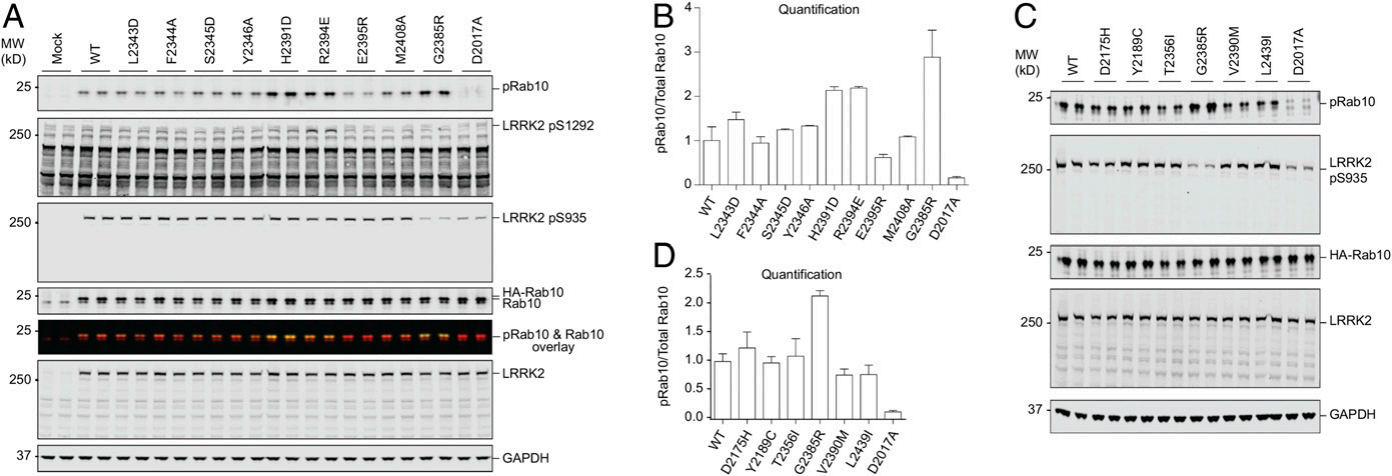
Effect of LRRK2 WD40 dimerization mutations and disease mutations on LRRK2 kinase activity. (*A*) HEK293 cells were transfected with the indicated WT and mutant LRRK2 variants together with either HA-empty vector (Mock) or HA-tagged Rab10. Twenty-four hours after transfection, cells were lysed and analyzed by immunoblotting with the indicated antibodies. From top to bottom: Rab10 phosphorylation at T73 (pRab10), LRRK2 autophosphorylation at S1292 (pS1292), LRRK2 phosphorylation at S935 (pS935), total HA-Rab10 and endogenous Rab10, pRab10 and Rab10 overlay, total LRRK2, and the loading control GAPDH. D2017A corresponds to the kinase-inactive LRRK2 mutant. Duplicated results are shown. (*B*) Quantification of pRab10 over total Rab10 in *A*. The WT ratio is set at 1.0. Errors indicated mean ± STD for three independent experiments. (*C*) HEK293 cells were transfected with the indicated WT and mutant LRRK2 variants together with HA-tagged Rab10. Twenty-four hours after transfection, cells were lysed and analyzed by immunoblotting with the indicated antibodies. From top to bottom: Rab10 phosphorylation at T73 (pRab10), LRRK2 phosphorylation at S935 (pS935), total HA-Rab10, total LRRK2, and the loading control GAPDH. D2017A corresponds to the kinase-inactive LRRK2 mutant. Duplicated results are shown. (*D*) Quantification of pRab10 over total Rab10 in *C*. The WT ratio is set at 1.0. Errors indicated mean ± STD for three independent experiments.

The Rab family GTPases belonging to the Ras small GTPase superfamily are important kinase substrates of LRRK2 (39), which localize to exocytic and endocytic compartments to regulate intracellular vesicle trafficking (40). It has been shown that LRRK2 directly phosphorylates a conserved Thr or Ser residue in the effector-binding switch II motifs of Rab proteins, which inhibits the binding to the Rab GDP-dissociation inhibitor factors that are required for membrane delivery and recycling (39). To investigate the functional effect of LRRK2 WD40 dimerization, we assessed the LRRK2 kinase activity using Rab10 phosphorylation on residue T73 using a recently elaborated specific anti-phospho-Rab10 antibody (41), upon cotransfection of WT and mutant LRRK2 with Rab10 in HEK293 cells (42) (Fig. 4*A* and *SI Appendix*, Table S3). LRRK2 autophosphorylation on S1292 (43) and phosphorylation at the well-studied S935 biomarker site (44) were also determined. We found that mutations at the dimerization interface, H2391D, R2394E, and the disease-related G2385R, moderately enhanced Rab10 phosphorylation by about twofold, while the remaining mutants L2343D, F2344A, S2345D, Y2346A, E2395R, and M2408A did not significantly impact Rab10 phosphorylation (Fig. 4*A*). As a control, the kinase-inactive LRRK2 mutant D2017A did not induce Rab10 phosphorylation. These results were confirmed by quantification on the ratio of pRab10 phosphorylation over total Rab10 for three independent experiments (Fig. 4*B*).

Because R1441G and G2019S are two recurrent PD variants, we also generated the same sets of LRRK2 WD40 domain mutations in these backgrounds. In the R1441G background, addition of the LRRK2 WD40 domain mutations did not further alter Rab10 phosphorylation (*SI Appendix*, Fig. S4 *A* and *B*). In contrast, in the G2019S background, the LRRK2 WD40 domain mutations exerted similar effects as in the WT background, with H2391D, R2394E, and the disease-related G2385R moderately increasing Rab10 phosphorylation (*SI Appendix*, Fig. S4 *B* and *C*). It has been shown previously that G2019S mutation alone does not change Rab10 phosphorylation (45). Only the R2394E mutation consistently promoted LRRK2 phosphorylation on S1292 above background, an event that is thought to be autophosphorylation (43) (Fig. 4*A* and *SI Appendix*, Fig. S4 *A*–*C*). Of note, previous studies reported that G2385R did not lead to an obvious change in autophosphorylation (46) but clearly led to an increase in Rab10 transphosphorylation (47). In contrast, phosphorylation on LRRK2 S935, which is thought to be regulated by LRRK2 via potentially other kinases (44), was interestingly reduced for the disease-related G2385R mutant as well as the kinase-inactive mutant D2017A, both in the WT and the G2019S backgrounds (Fig. 4*A* and *SI Appendix*, Fig. S4 *B* and *C*). Consistent with previous work, the R1441G mutation considerably suppressed Ser935 phosphorylation (44). For LRRK2 WD40 mutations in the R1441G background, no obvious changes in S935 phosphorylation were observed except slight enhancement for the kinase-inactive mutant D2017A (*SI Appendix*, Fig. S4 *A* and *B*). We also investigated the remaining WD40 domain disease-associated variants on LRRK2 kinase activity using Rab10 phosphorylation. In contrast to G2385R, these variants did not cause a significant difference in Rab10 phosphorylation (Fig. 4 *C* and *D* and *SI Appendix*, Fig. S4*D* and Table S3).

Enhanced LRRK2 kinase activity for some of the WD40 dimerization defective mutants, including H2391D, R2394E, and the disease variant G2385R, indicates that WD40 dimerization may be inhibitory to the kinase activity. However, there is no strict correlation between dimerization and LRRK2 kinase activity, suggesting that dimerization is not the only factor in the regulation of this complex enzyme.

## Discussion

Despite the biological and clinical importance of LRRK2, limited structural information is currently available. Our WD40 domain structure contributes to improving this situation by adding a high-resolution view of a second domain of human LRRK2 after the previously determined structure of the ROC domain of human LRRK2 (9). We found that the WD40 domain forms dynamic dimers in solution, and both structure-based mutations and PD-associated disease variants in the WD40 domain mainly impair its dimerization. Hence, our structure provides a template for elucidating the biological function of the WD40 domain and for understanding how mutations of the domain may affect the function of LRRK2.

Our structural and functional analysis here only touched the surface of the complexity of LRRK2 biology. For one, dimerization in the WD40 domain does not have a strict correlation with LRRK2 kinase activity, as determined by Rab10 phosphorylation. Only three WD40 dimerization-defective mutants enhanced LRRK2 kinase activity by about twofold, while the other mutants did not show an obvious effect. One possible scenario is that WD40 domain dimerization does play a role in LRRK2 biological and pathological function but is not sufficient on its own. Interestingly, in the low-resolution full-length, active LRRK2 structure model, the WD40 domain is not adjacent to each other for dimer formation (13, 14), suggesting that WD40 domain dimerization may be inhibitory. This is puzzling as the kinase activity of full-length LRRK2 does appear to reside with dimers (48), while GTPase activity of the ROC domain is equivalent in dimers and monomers (49). Is it possible that there are at least two different dimerization states of full-length LRRK2? A WD40 domain-mediated dimer may be important for other interactions by LRRK2 such as with microtubules (19). In fact, it is unknown whether the WD40 domain mutations generated here disrupt the dimerization of full-length LRRK2.

A completely different scenario is that the surface on blade V, identified by mutants H2391D, R2394E, and the disease-related G2385R, or the conserved top surface on blade III and IV (Fig. 1*D*), may be important for partner interactions by the WD40 domain (Fig. 2*A* and *SI Appendix*, Fig. S2). The blade V surface partially overlaps with the WD40 dimerization interface, and it is likely that this surface, as well as the conserved top surface, is used for additional intramolecular or intermolecular interactions to regulate LRRK2 function.

While further studies will be required to dissect these scenarios to elucidate the exact role of the WD40 domain and its dimerization in the function of LRRK2, our data clearly showed moderately increased LRRK2 kinase activity for the PD-associated G2385R variant. Hence, patients with WD40 mutations that are relatively common in certain Asian populations might benefit from receiving LRRK2 kinase inhibitors. These data also support the general concept that like all other LRRK2 pathogenic mutations, WD40 mutations also result in activation of LRRK2 and promote Rab10 phosphorylation.

## Materials and Methods

### Protein Expression and Purification.

Human LRRK2 WD40 domain (residues 2142–2527) was expressed in Sf9 insect cells via baculovirus infection and purified to homogeneity.

### Crystallization and Structure Determination.

The LRRK2 WD40 domain was crystallized using hanging drop vapor diffusion. The structure was determined by anomalous diffraction phasing and refined to 2.6-Å resolution. The atomic coordinates and structure factors have been deposited in the Protein Data Bank, www.wwpdb.org [PDB ID codes 6DLO (50) and 6DLP (51)].

### MALS.

To measure the molecular mass of the WD40 domain protein in solution, we used a three-angle light scattering detector and a refractive index detector, which were coupled to a chromatography system.

### Quantitative Immunoblot Analysis.

Proteins were electrophoretically transferred onto nitrocellulose membrane. After incubation with antibodies, protein bands were acquired via near infrared fluorescent detection using Odyssey CLx imaging.

## Supplementary Material

Supplementary File
