# Supplementary material for: Crystal structure of the WD40 domain dimer of LRRK2
Source: Proc Natl Acad Sci U S A. 2019 Jan 11;116(5):1579–84. doi: 10.1073/pnas.1817889116 (PMC6358694; doi:10.1073/pnas.1817889116)
Supplement: Supplementary File [file pnas.1817889116.sapp.pdf]

Supporting Information

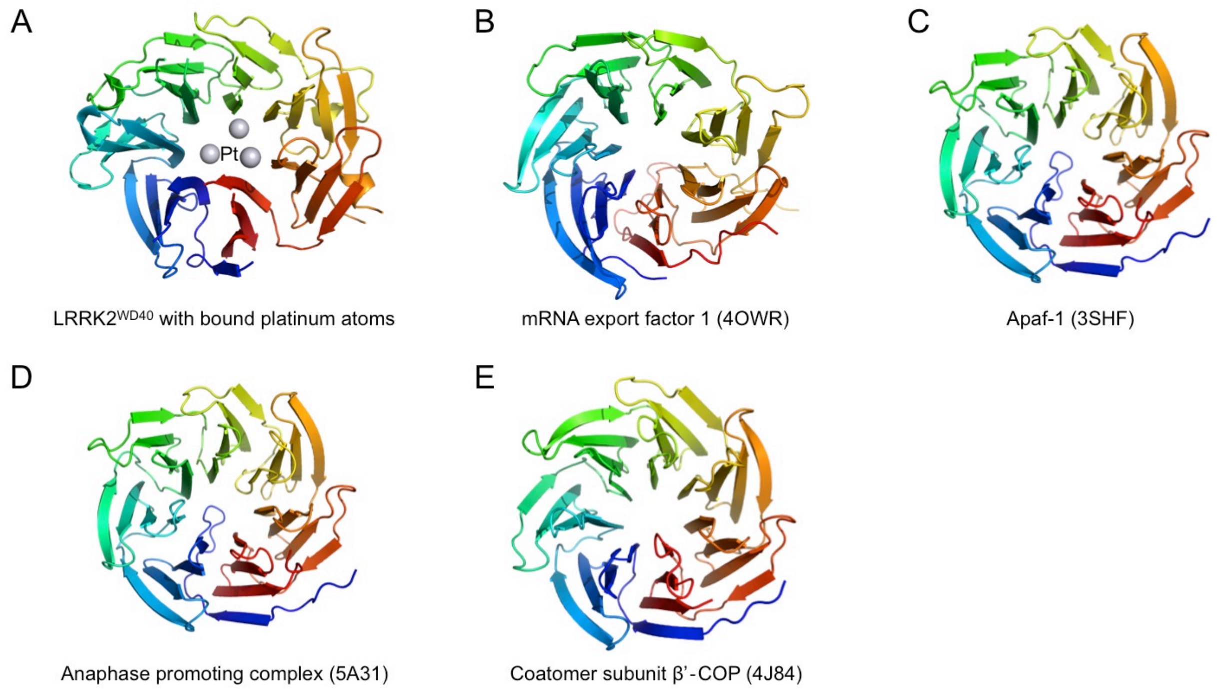

Fig. S1. Structural overview. (A) Bound platinum atoms in the LRRK2 WD40 domain structure. (B-E) Top matches from structural homology search by DALI (1).

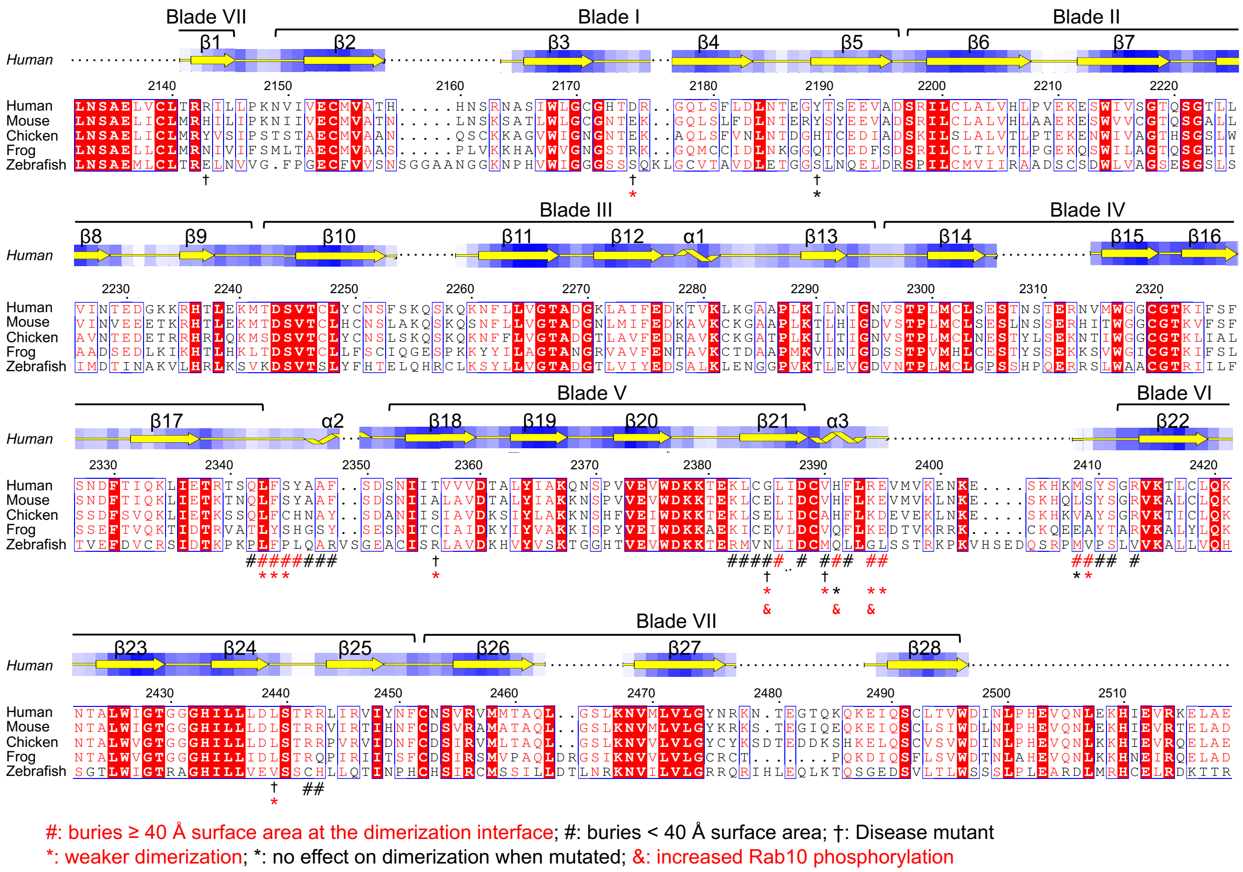

Fig. S2. Sequence alignment among LRRK2 proteins from several species, showing secondary structures and locations of the blades. Residues involved in the dimerization, disease mutations and the effects of these mutations in Rab10 phosphorylation are marked.

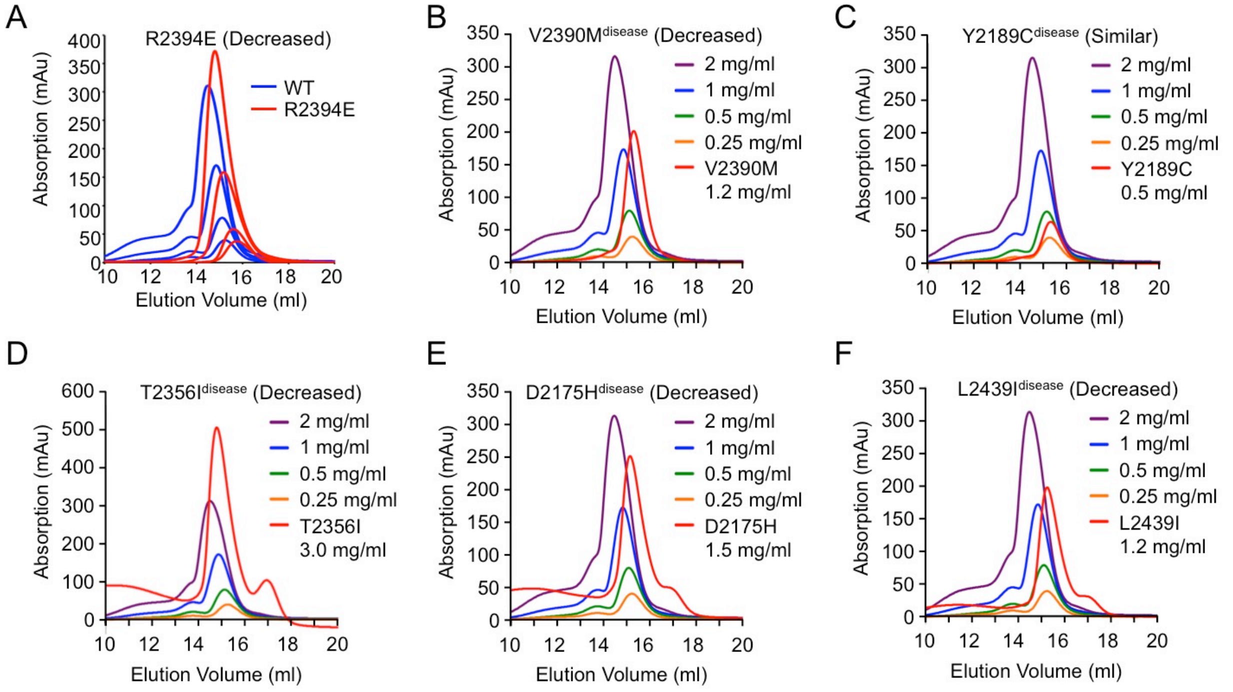

Fig. S3. Effects of structure-based and disease mutations on LRRK2 WD40 domain dimerization. (A) WT (blue) and R2394E (red) were subjected to gel filtration chromatography at 2, 1, 0.5 and 0.25 mg/ml. (B-F) Gel filtration profile of a WD40 domain mutant at one concentration superimposed with that of a WT WD40 domain at four indicated concentrations.

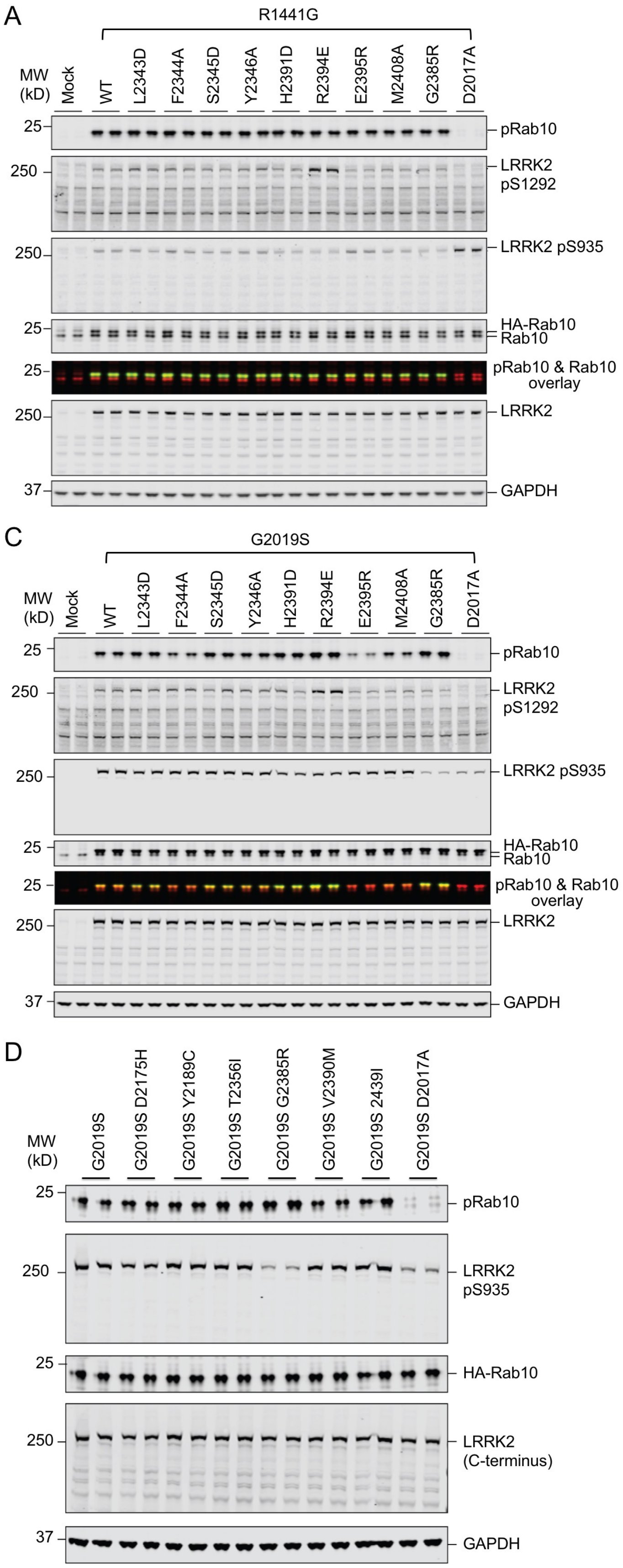

A
R1441G
MW (kD)
Mock
WT
L2343D
F2344A
S2345D
Y2346A
H2391D
R2394E
E2395R
M2408A
G2385R
D2017A
25
250
250
25
25
250
37
pRab10
LRRK2 pS1292
LRRK2 pS935
HA-Rab10
Rab10
pRab10 & Rab10 overlay
LRRK2
GAPDH
B
R1441G
G2019S
WT
Mock
H2391D
R2394E
G2385R
D2017A
WT
H2391D
R2394E
G2385R
D2017A
WT
H2391D
R2394E
G2385R
D2017A
pRab10
LRRK2 pS1292
LRRK2 pS935
HA-Rab10
Rab10
pRab10 & Rab10 overlay
LRRK2
GAPDH
C
G2019S
MW (kD)
Mock
WT
L2343D
F2344A
S2345D
Y2346A
H2391D
R2394E
E2395R
M2408A
G2385R
D2017A
25
250
250
25
25
250
37
pRab10
LRRK2 pS1292
LRRK2 pS935
HA-Rab10
Rab10
pRab10 & Rab10 overlay
LRRK2
GAPDH
D
MW (kD)
G2019S
G2019S D2175H
G2019S Y2189C
G2019S T2356I
G2019S G2385R
G2019S V2390M
G2019S 2439I
G2019S D2017A
25
250
25
250
37
pRab10
LRRK2 pS935
HA-Rab10
LRRK2 (C-terminus)
GAPDH

Fig. S4. Effect of LRRK2 WD40 dimerization mutations and disease mutations on LRRK2 kinase activity. (A-C) Measurements of LRRK2 kinase activity in the background of R1441G (A-B) and G2019S (B-C). HEK293 cells were transfected with the indicated wild-type (WT) and mutant LRRK2 variants together with either HA-empty vector (Mock) or HA-tagged Rab10. 24 hours post-transfection, cells were lysed and analyzed by immunoblotting with the indicated antibodies. From top to bottom: Rab10 phosphorylation at T73 (pRab10), LRRK2 autophosphorylation at S1292 (pS1292), LRRK2 phosphorylation at S935 (pS935), total HA-Rab10, pRab10 and Rab10 overlay, total LRRK2 and the loading control GAPDH. D2017A corresponds to the kinase-inactive LRRK2 mutant. Duplicated results are shown. (D) Selective Parkinson's disease associated mutants in the background of G2019S. Experiments were performed similarly as in (A-C).

Table S1. Heavy atom compounds used for search of derivatives

Heavy atom compounds (1 mM in the cryo-solution)	Crystals survived	Anomalous signal
Potassium tetrachloroplatinate (II)	No	
Potassium tetracyanoplatinate (II)	Yes	No
Potassium tetrabromoplatinate (II)	Yes	No
Potassium hexabromoplatinate (IV)	Yes	No
Platinum (II) bis (ethylene-diamine) chloride	No	
Potassium hexachloroplatinate (IV)	Yes	No
Trans-platinum (II) diammine dichloride	Yes	Yes
cis- platinum (II) diammine dichloride	Yes	No
Mersalyl acid	Yes	No
4-Chloromercuribenzoic acid	Yes	No
Mercury (II) acetate	Yes	No
Mercury (I) chloride	Yes	No
Mercury (II) chloride	No	
Mercury (II) nitrate monohydrate	No	
Mercury (II) iodide	No	
4-(chloro mercuri)benzenesulfonic acid	No	
Mercurochrome	No	

Table S2. Crystallographic statistics

	Native	Trans-platinum (II) diammine dichloride
Date collection		
Space group	$P2_12_12_1$	$P2_12_12_1$
Cell dimensions: a, b, c (Å) / α, β, γ (°)	79.0, 103.1, 113.6 /90.0, 90.0, 90.0	79.3, 103.6, 112.7 /90.0, 90.0, 90.0
Wavelength (Å)	0.98	
Resolution (Å)	2.62	3.45
Total reflections		
Unique reflections		
Completeness (%)	97.1	95.8
I/σ	17.5	7.6
R_{merge} (%)	0.138	0.159
Multiplicity	13.9	6.1
Refinement		
R_{work} / R_{free} (%)	0.2342 / 0.2753	
RMSD bond length (Å)	0.002	
RMSD bond angles (°)	0.612	
Ramachandran favored / outliers (%)	95.78 / 0.17	
Average B-factor ($Å^2$)	37.8	
Molprobity score	1.71	
Molprobity clash score (percentile)	7.57 (98th)	

Table S3. LRRK2^{WD40} dimerization and disease mutants and their effects on Rab10 phosphorylation

Relative to WT	Dimerization	pRab10	pRab10/with G2019S	pRab10/with R1441G
R2143H^{disease}	Poor Exp.	N.D.	N.D.	N.D.
D2175H^{disease}	Decreased	Similar	Similar	Similar
Y2189C^{disease}	Similar	Similar	Similar	Similar
L2343D	Decreased	Similar	Similar	Similar
F2344A	Decreased	Similar	Similar	Similar
S2345D	Decreased	Similar	Similar	Similar
Y2346A	Decreased	Similar	Similar	Similar
T2356I^{disease}	Decreased	Similar	Similar	Similar
G2385R^{disease}	Decreased	Increased	Increased	Similar
D2388K	Poor Exp.	N.D.	N.D.	
V2390M^{disease}	Decreased	Similar	Similar	Similar
H2391D	Poor Exp.	Increased	Increased	Similar
R2394E	Decreased	Increased	Increased	Similar
E2395R	Decreased	Similar	Similar	Similar
M2408A or M2408E	Similar	Similar	Similar	Similar
S2409A	Decreased	N.D.	N.D.	N.D.
L2439I^{disease}	Decreased	Similar	Similar	Similar
D2017A$^{kinase\text{-}inactive}$	N.D.	None	None	None

N.D: Not done

Materials and Methods

General Methods. DNA constructs were amplified in DH5α strain of E. coli and purified using Hi-Speed Plasmid Maxi Kit from Qiagen (Cat# 12663). DNA cloning procedures were undertaken using standard protocols. Sequences of DNA constructs used in the present study were verified using Genewiz (http://www.genewiz.com) and the Sequencing Services (http://www.dnaseq.co.uk).

Protein Expression and Purification. Human LRRK2 WD40 domain (residues 2142-2527) was cloned into a reconstructed pFastBac1 vector with N-terminal 6xHis and maltose binding protein (MBP) and a *tobacco* etch virus (*TEV*) protease cleavage site. The protein was expressed in Sf9 insect cells via baculovirus infection. The cell pellets were lysed by sonication and the lysates were spun down by ultracentrifugation. The protein was affinity purified from the supernatants by amylose resin, and the eluted fractions were concentrated and further purified by gel filtration chromatography using a Superdex 200 (10/300 GL) column (GE Healthcare). The His-MBP tag was removed by TEV and the cleaved WD40 domain protein was separated from the tag by a heparin affinity column (GE Healthcare).

Crystallization and Structure Determination. The LRRK2 WD40 protein was concentrated to 9 mg/ml in a buffer containing 20 mM Tris-HCl at pH 7.5, 150 mM NaCl and 1 mM tris(2-carboxyethyl)phosphine (TCEP) and crystallized using hanging drop vapor diffusion. The best crystals appeared under the condition of 0.1 M Tris-HCl at pH 8.5, 1 M LiCl, 16% polyethylene glycol (PEG) 6000, and 10% additive of 30% galactose. Thirteen heavy atom compounds were screened at different conditions for obtaining derivatives (Table S1). One WD40 domain crystal that was soaked in 1 mM trans-platinum (II) diammine dichloride for 1.5 hours gave diffraction to 3.45 Å resolution and was used for phase determination by single wavelength anomalous diffraction (SAD) calculations in Phenix (2). A native diffraction data at up to 2.6 Å resolution were collected. All diffraction data were acquired at the NE-CAT beam line of the Advanced Photo Source (APS) and processed with XDS (3). The atomic model was built in Coot (4) and refined in Phenix (2). The final R_{work} and R_{free} are 0.23 and 0.28 (Table S2).

Multi-Angle Light Scattering (MALS). To measure the molecular mass of the WD40 domain protein in solution, we used a three-angle light scattering detector (mini-DAWN TRISTAR) and a refractive index detector (Optilab DSP) (Wyatt Technology), which were coupled to a chromatography system. We injected the protein into a Superdex 200 (10/300 GL) gel filtration column (GE Healthcare) equilibrated in a buffer containing 20 mM Tris-HCl at pH 7.5 and 150 mM NaCl, 1mM TCEP. Data were collected every 0.5 s at a flow rate of 0.2 mL/min. Data analysis was carried out using ASTRA V.

Antibodies. Rabbit monoclonal antibody for LRRK2 phospho-Ser935 (UDD2) (5, 6) was purified at the University of Dundee and used at 1 µg/ml final concentration. Mouse monoclonal antibody against total LRRK2 (C-terminus) was from Antibodies Incorporated (Cat# 75-253) and used to multiplex with LRRK2 pS935 antibody at 1:1000 dilution. Rabbit monoclonal antibody for 9phosphor-Rab10 (T73) [MJF-R21] was from Abcam (Cat# ab230261) and used at 0.5 µg/ml final concentration. A mouse monoclonal antibody raised against human recombinant Rab10 (sequence 100% identical to mouse) was generated by Nanotool Antibodies (http://www.nanotools.de/) to enable LI-COR multiplexing of the phosphor-Rab10 rabbit monoclonal antibody with a total Rab10 antibody (PMID 29127256). The MJFF-total Rab10 mouse monoclonal antibody was at 0.5 µg/ml final concentration. Mouse anti-glyceraldehyde-3-phosphate dehydrogenase (GAPDH) monoclonal antibody was from Santa Cruz Biotechnology (Cat# sc-32233) and used at 1:5000 dilution. Goat anti-mouse IRDye 800CW (Cat# 926-32210)

and IRDye 680LT (Cat# 926-68020), goat anti-rabbit IRDye 800CW ((Cat# 926-32211) secondary antibodies were from LI-COR and used at 1:10000 dilution.

Plasmids. The following constructs were used for LRRK2 cellular assays: HA-Rab10 WT (DU44250); Flag-tagged full length LRRK2 WT and mutants L2343D, F2344A, S2345D, Y2346A, H2391D, R2394E, E2395R, M2408A, G2385R, D2017A, D2388R, D2388K, D2175H, Y2189C, T2356I, V2390M, and L2439I, (DU6841, DU27375, DU27376, DU27377, DU27378, DU27446, DU27379, DU27412, DU27380, DU27381, DU10128, DU62075, DU62052, DU62374, DU62391, DU62375, DU62376, DU62393); Flag-tagged full length LRRK2 mutants G2019S, G2019S+L2343D, G2019S+F2344A, G2019S+S2345D, G2019S+Y2346A, G2019S+H2391D, G2019S+R2394E, G2019S+E2395R, G2019S+M2408A, G2019S+G2385R, G2019S+D2017A, G2019S+D2388R and G2019S+D2388K (DU10129, DU27382, DU27394, DU27395, DU27383, DU27384, DU27385, DU27386, DU27396, DU27387, DU52723, DU62076/DU62053); Flag-tagged full length LRRK2 mutants R1441G, R1441G+L2343D, R1441G+F2344A, R1441G+S2345D, R1441G+Y2346A, R1441G+H2391D, R1441G+R2394E, R1441G+E2395R, R1441G+M2408A, R1441G+G2385R, R1441G+D2017A, R1441G+D2388R and R1441G+D2388K (DU13077, DU27388, DU27389, DU27390, DU27397, DU27391, DU27405, DU27392, DU27398, DU27393, DU52702, DU62077, DU62062). All cDNA clones generated for the present study can be requested via MRC-PPU Reagents and Services website (https://mrcppureagents.dundee.ac.uk).

Cell Culture, Transfection, and Lysis. HEK293 cells were cultured in Dulbecco's modified Eagle's medium containing 10% fetal bovine serum, penicillin (100 U/ml)/ streptomycin (100 μg/ml), and 2 mM glutamine. Cells were transfected with polyethylenimine HCl MAX 4000 (Poly sciences, Inc.) as described previously (7). In brief, 0.4 μg HA-tagged Rab10 construct was mixed with 1.6 μg Flag-tagged LRRK2 construct in 0.5 ml of OPTI-MEM media for transfecting each well of a 6-well plate. The transfection mix was incubated at RT for 5 min then added onto 70% confluent HEK293 cells subcultured one day prior to transfection. Cells were lysed 24 hours after transfection in ice-cold lysis buffer containing 50 mM Tris-HCl, pH 7.5, 1% (v/v) Triton X-100, 1 mM EGTA, 1 mM sodium orthovanadate, 50 mM NaF, 0.1% (v/v) 2-mercaptoethanol, 10 mM 2-glycerophosphate, 5 mM sodium pyrophosphate, 1 µg/ml mycrocystin-LR (Enzo Life Sciences), 270 mM sucrose, and Complete EDTA-free protease inhibitor cocktail (Roche). Lysates were cleared via centrifugation at 20,800 g for 15 min at 4°C, and supernatants were quantified by Bradford assay (Thermo Scientific) and subjected to immunoblot analysis. HEK293 cells used in this study were tested for mycoplasma contamination and confirmed as negative for experimental analysis.

Quantitative Immunoblot Analysis. Mix cell lysates with 4× SDS-PAGE loading buffer [250 mM Tris-HCl, pH 6.8, 8% (w/v) SDS, 40% (v/v) glycerol, 0.02% (w/v) Bromophenol Blue and 4% (v/v) 2-mercaptoethanol] to a final total protein concentration of 2 µg/µl and heated at 70 °C for 10 min. Load 20 µg HEK293 samples on to NuPAGE 4–12% Bis-Tris Midi Gel (Thermo Fisher Scientific, Cat# WG1403BOX) for electrophoresis at 130 V for 2 h with the NuPAGE MOPS SDS running buffer (Thermo Fisher Scientific, Cat# NP0001-02). Proteins were electrophoretically transferred onto nitrocellulose membrane (GE Healthcare, Amersham Protran Supported 0.45 µm NC) at 100 V for 90 min on ice in the transfer buffer [48 mM Tris-HCl and 39 mM glycine] at the end of electrophoresis. Transferred membrane was blocked at room temperature for 1 h with 5% (w/v) skim milk powder dissolved in TBS-T [20 mM Tris-HCl, pH 7.5, 150 mM NaCl and 0.1% (v/v) Tween 20]. Membranes were then incubated with indicated primary antibodies. After overnight incubation under rotation at 4°C, membranes were washed three times with TBS-T for 10 min each prior to secondary antibody incubation at room temperature for 1 h. Membranes were washed with TBS-T for three times with a 10 min

incubation for each wash after secondary antibody incubation. Protein bands were acquired via near infrared fluorescent detection using Odyssey CLx imaging.

References

1. Holm L & Sander C (1995) Dali: a network tool for protein structure comparison. *Trends Biochem. Sci.* 20:478-480.
2. Adams PD*, et al.* (2010) PHENIX: a comprehensive Python-based system for macromolecular structure solution. *Acta Crystallogr D Biol Crystallogr* 66(Pt 2):213-221.
3. Kabsch W (2010) Xds. *Acta Crystallogr D Biol Crystallogr* 66(Pt 2):125-132.
4. Emsley P & Cowtan K (2004) Coot: model-building tools for molecular graphics. *Acta Crystallogr D Biol Crystallogr* 60(Pt 12 Pt 1):2126-2132.
5. Dzamko N*, et al.* (2012) The IkappaB kinase family phosphorylates the Parkinson's disease kinase LRRK2 at Ser935 and Ser910 during Toll-like receptor signaling. *PLoS One* 7(6):e39132.
6. Davies P*, et al.* (2013) Comprehensive characterization and optimization of anti-LRRK2 (leucine-rich repeat kinase 2) monoclonal antibodies. *Biochem J* 453(1):101-113.
7. Reed SE, Staley EM, Mayginnes JP, Pintel DJ, & Tullis GE (2006) Transfection of mammalian cells using linear polyethylenimine is a simple and effective means of producing recombinant adeno-associated virus vectors. *J Virol Methods* 138(1-2):85-98.
